# Analogical and Digital Workflow in the Design and Preparation of the Emergence Profile of Biologically Oriented Preparation Technique (BOPT) Crowns over Implants in the Working Model

**DOI:** 10.3390/jcm8091452

**Published:** 2019-09-12

**Authors:** Guillermo Cabanes-Gumbau, David Soto-Peñaloza, Miguel Peñarrocha-Diago, María Peñarrocha-Diago

**Affiliations:** 1 Master’s program in Oral Surgery and Implant Dentistry, Stomatology Department, Faculty of Medicine and Dentistry, University of Valencia, 46010 Valencia, Spain; 2 Professor and Chairman of Oral Surgery, Stomatology Department, Faculty of Medicine and Dentistry, University of Valencia, 46010 Valencia, Spain; 3 Full Professor of Oral Surgery, Stomatology Department, Faculty of Medicine and Dentistry, University of Valencia, 46010 Valencia, Spain

**Keywords:** BOPT implants, BOPT technique, emergency profile, analogical workflow, digital workflow, digital dentistry, soft tissues

## Abstract

The Biologically Oriented Preparation Technique (BOPT), developed by Ignacio Loi and published in 2008, is a consolidated concept in the modeling and preservation of pericoronal soft tissues. The present study describes the analogical and digital methods allowing adequate design and preparation of the emergence profile of BOPT crowns in the working model, with a view to comparing the workflow and advantages of each method. At present, not all the digital procedures have been fully optimized to completely replace the traditional analogical methods. Nevertheless, it is only a matter of time until dental digitalization technology totally replaces the analogical clinical methods. The digital workflow for this procedure is quickly implemented and optimized, and represents the most realistic option, with possibilities for further development in the immediate future.

## 1. Introduction

The Biologically Oriented Preparation Technique (BOPT) was developed by Ignacio Loi and published in 2008, and has become a consolidated concept in the modeling and preservation of pericoronal soft tissues [1,2,3,4]. A recent prospective clinical study showed that restorations placed on teeth prepared with the BOPT approach present good periodontal behavior, an increase of gingival thickening and stability of marginal tissue after 4 years of follow-up, showing lower rates of, both, biological and technical complications [5].

More recently, this philosophy has been transferred to implantology in the preparation of crowns (with certain specific features) over truncoconical implant-supported abutments without a finishing line and convergent towards the occlusal plane. This novel approach demonstrated a significant increase in the thickening of the peri-implant soft tissue volume after 10 months of prosthetic loading in a recent pilot clinical study [6].

**Figure 1:**
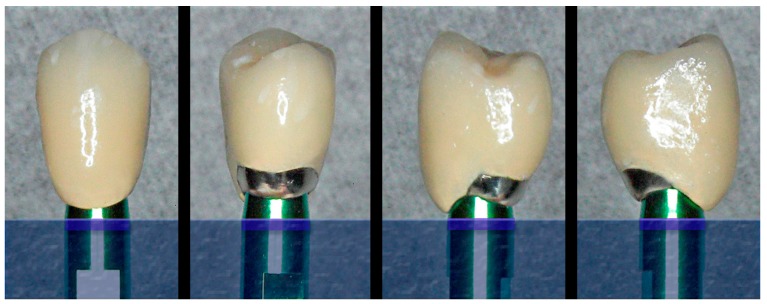
The cervical design of the implant and abutment, with shoulder-less and convergent morphology, allows the personalization of the emergence profile of the BOPT crown, with over-contouring and/or festooning at different levels at each of the surfaces of the prosthesis.

**Figure 2:**
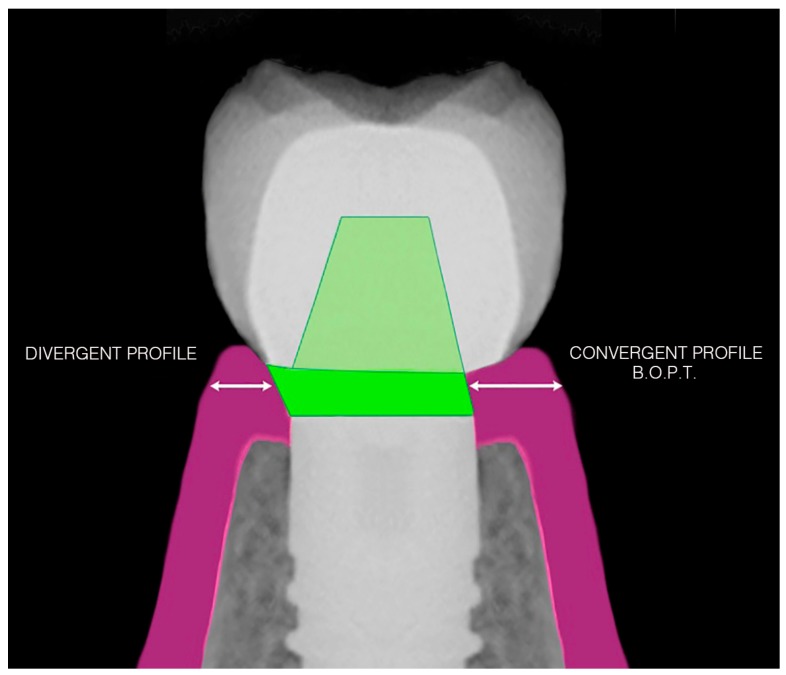
The difference in cervical design with a divergent or convergent BOPT profile conditions greater available space for the pericoronal mucosal sealing tissue on the right side.

One of the key principles of the BOPT technique is that gingival tissue is able to adapt to prosthetic shape in both prostheses over teeth and over implants. Accordingly, adequate design of cervical emergence of the crown plays a crucial role in the application of this technique (buccolingual over-contouring and inter-proximal festooning) along with the use of shoulder-less convergent abutments that are less voluminous than conventional abutments in the “subcritical profile” zone [7]. These characteristics favor both the supracrestal tissue attachment [8] and peri-implant mucosal sealing through collagen fibers that remain stable and thick over time, with greater efficacy in protecting the supporting bone tissues [5,9,10] (Figure 1 and Figure 2). 

The present study describes analogical and digital methods allowing adequate design and preparation of the emergence profile of BOPT crowns in the working model, as an essential part of the development of implant-supported crowns of this type.

## 2. Description of the Technique

A description is provided below of the preparation of cervical emergence for BOPT prostheses over implants in the working model, based on two workflow options: analogical and digital. Both protocols involve the use of a plaster (analogical) or resin prototype working model (digital) on which the pre-existing peri-implant gingival morphology has been “remodeled—modified” to obtain a gingival emergence design optimized for the prosthesis, which is then manufactured in a “guided” manner from that “starting point.” In other words, the mucosal emergence design artificially created in the working model around the analogical-abutment unit will simulate the desired final cervical morphology, which is then transferred by the laboratory technician to the crown (provisional or definitive) that will be prepared adapted to that specific cervical design [11,12,13].

## 3. Analogical Workflow

**Figure 3:**
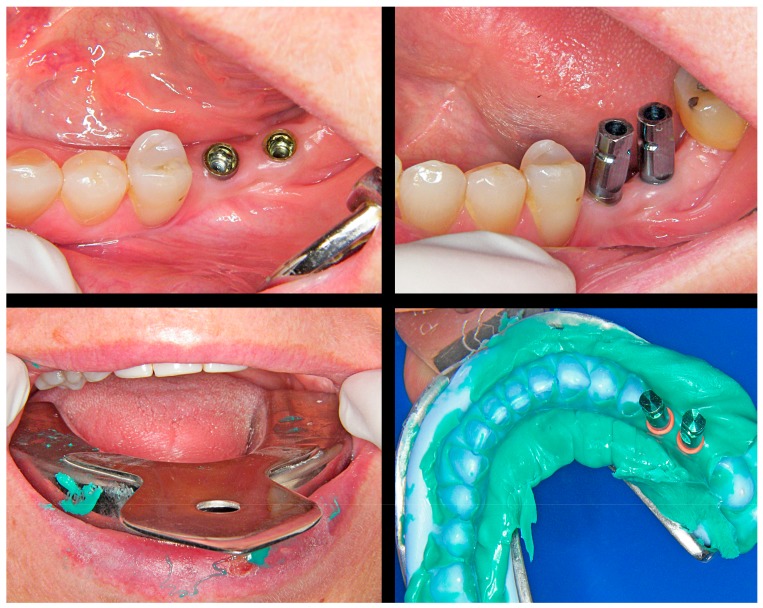
Silicone impression and placement of an O-ring spacer around the head of each analog before casting.

**Figure 4:**
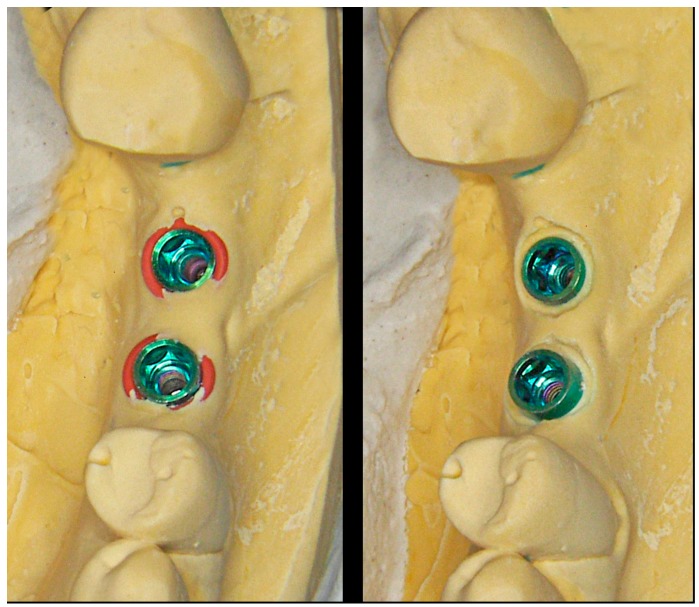
The O-ring affords a small free space around the coronal portion of the analog. This will facilitate the selective reduction of the plaster model without deteriorating it.

After obtaining the silicone impression (with an open or closed cuvette), and before casting, we insert a silicone O-ring (measuring about 1 mm in thickness) around the head of each analog (Figure 3), with the purpose of creating a small plaster-free space in the mentioned zone (Figure 4). This will facilitate the preparation of the emergence profile through selective reduction of the model, without deteriorating the margins of the analog (located slightly subgingival) during the procedure.

**Figure 5:**
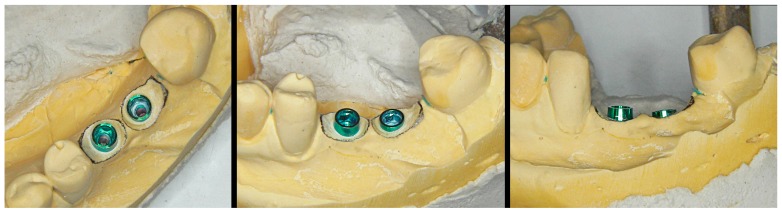
A pencil is used on the model to trace the cervical limits of the desired emergence profile of the crown.

**Figure 6:**
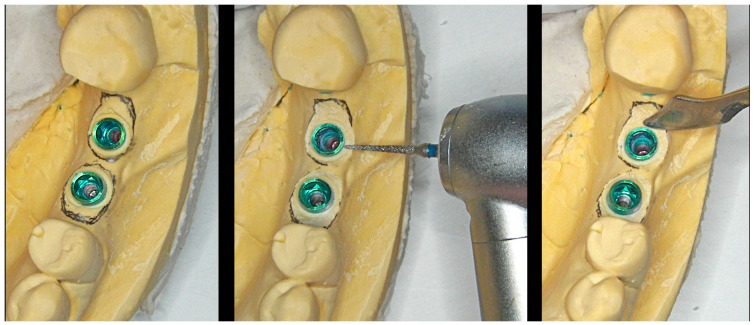
Manual reduction of the plaster using drills and laboratory scalpels.

Initially, a pencil is used on the model to trace the limits of the cervical contour of the desired emergence profile for each crown. A manual reduction of the plaster is performed (from the head of the analog to the traced limit) using diamond drills and laboratory scalpels, until the adequate “ovoid” emergence profile characteristic of BOPT crowns is obtained, with correct buccolingual over-contouring (to guide cervical crown morphology on the free surfaces) and adequate inter-proximal festooning (to maintain sufficient papilla thickness), without extending in depth within the sulcus more than 1–1.5 mm (Figure 5 and Figure 6).

In this way, the dentist or laboratory technician, by following the specific “clinical recipe” of each case, can design and produce a correct crown emergence profile through selective reduction of the plaster model, based on objective criteria according to the gingival thickness and radiographic bone profile.

**Figure 7:**
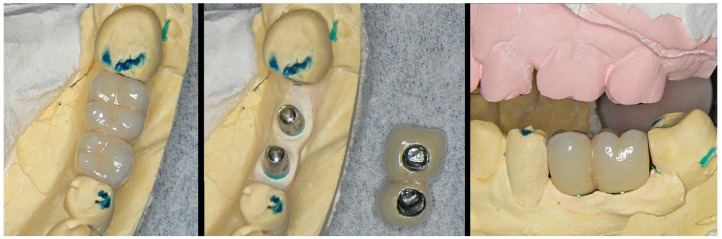
Ceramometallic crowns prepared and adapted to the emergence profile previously created in the model.

**Figure 8:**
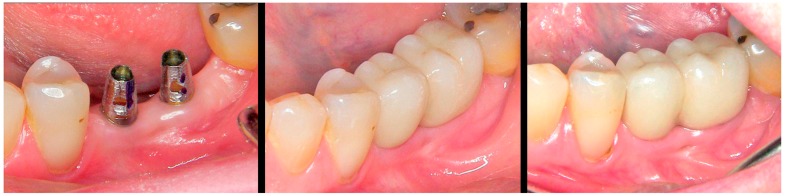
Intraoral situation. Left: truncoconical abutments without finishing line. Center: freshly cemented BOPT crowns. Right: view of the prosthesis and thickening of the pericoronal sealing soft tissues after 18 months.

The BOPT crowns produced over these personalized emergences in the model (Figure 7) will transfer the mentioned gingival morphology to the mouth of the patient, affording an adequate cervical crown profile from the time of placement of the prosthesis (through selective gingival compression). The outcome will moreover improve over time in terms of pink color aesthetics and the quality and thickness of the peri-implant mucosal sealing tissues (Figure 8).

## 4. Digital Workflow

The above-described procedure involving the plaster model is perfectly transferable to the digital setting thanks to intraoral scanning and the design and preparation of prototype models that incorporate from the start the coronary emergence profile specifically individualized for the BOPT prosthesis of each implant.

**Figure 9:**
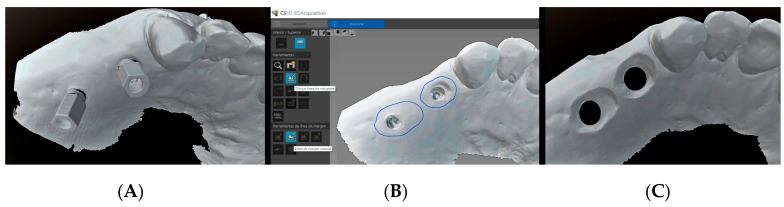
The dentist performs the intraoral scan (**A**) and uses the software of the clinical scanner to design the limits of the cervical contour of the desired emergence profile for each crown (**B**). In the laboratory, the technician designs the emergence profile from the neck of the implant-abutment unit to the contour line indicated by the clinician (**C**).

In this technique the dentist, after performing the intraoral scan and based on the software (CS IO 3D acquisition software (version 3.1.0.), Carestream Dental, Atlanta, GA, USA) of the clinical scanner (CS 3600^®^, Carestream Dental, Atlanta, GA, USA), can use the corresponding tool to trace the limits of the cervical contour of the desired emergence profile for each crown on the digital model (in a way similar to the pencil tracing of lines in the analogical technique) (Figure 9).

In this way the clinician prepares an adequate “digital recipe” specifically adapted to each case, and which will include the digital model files with the tracing of the cervical contour and written indication of the crown emergence starting point, from the head of the implant or from the abutment, specifying the required distance (in mm) in depth buccal, lingual, and inter-proximal (ideally not more than 1–1.5 mm).

**Figure 10:**
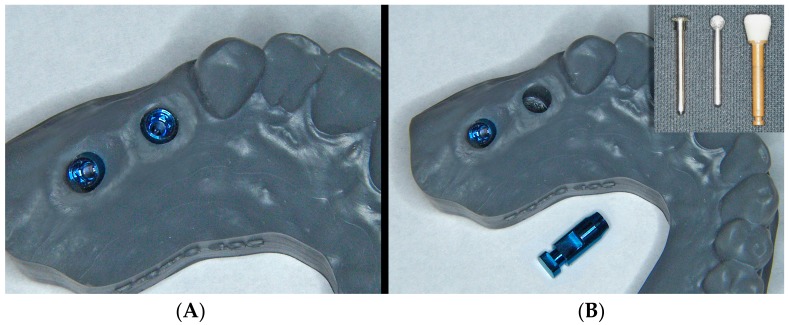
The prototype already includes the emergence profile previously designed with the computer (**A**). If necessary, subsequent modifications of the profile can be made using rotary instruments in a simple manner, extracting the analog (**B**).

**Figure 11:**
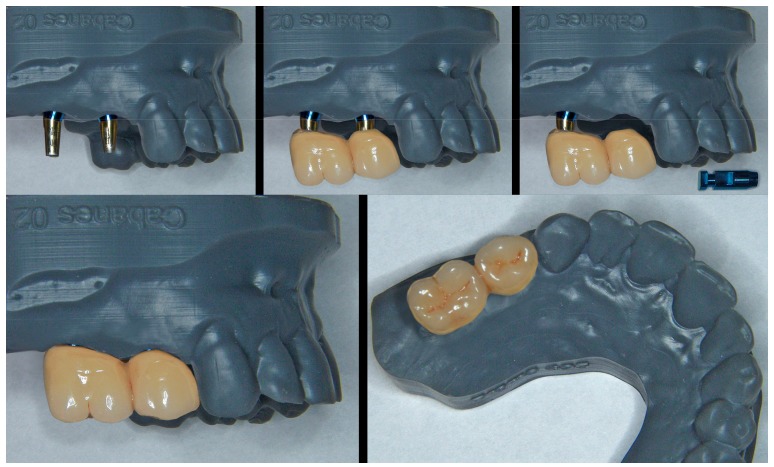
The metal-ceramic crowns are prepared with their cervical portion “guided” by the emergence morphology of the model. The possibility of extracting the analogues facilitates preparation and fitting of the ceramics to the contours of the prototype.

With this specific information, the laboratory can design upon the digital model (before printing) the morphology of the “reduction” around the implant required to achieve a personalized emergence profile (Figure 9), which is thus already modified at the time of manufacture of the prototype model. The prototype model incorporates information about soft tissue contour that allowing working with extractable analogs (Figure 10). The emergence profile adaptation consists of detecting friction points and interferences through insertion and extraction movements of the crowns in the model (Figure 11).

**Figure 12:**
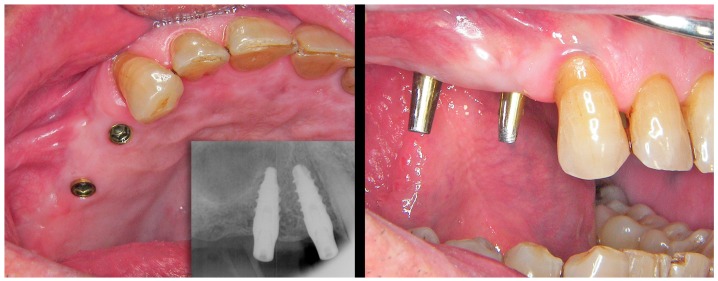
Intraoral situation of the implants and prosthetic abutments before placement.

**Figure 13:**
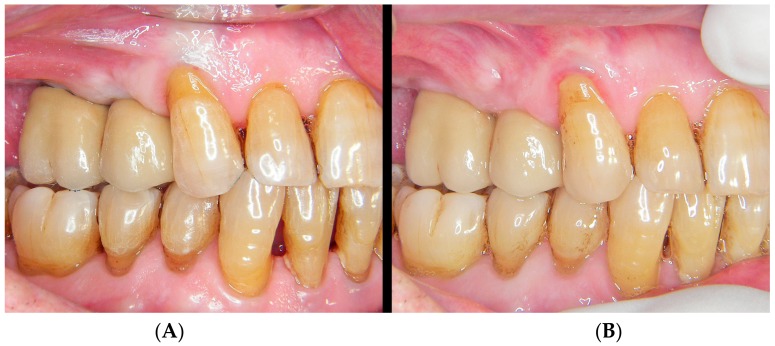
(**A**) freshly cemented prosthesis showing slight tissue ischemia as a result of controlled mucosal compression. (**B**) The view of the prosthesis and thickening of the pericoronal sealing soft tissues after 18 months.

**Figure 14:**
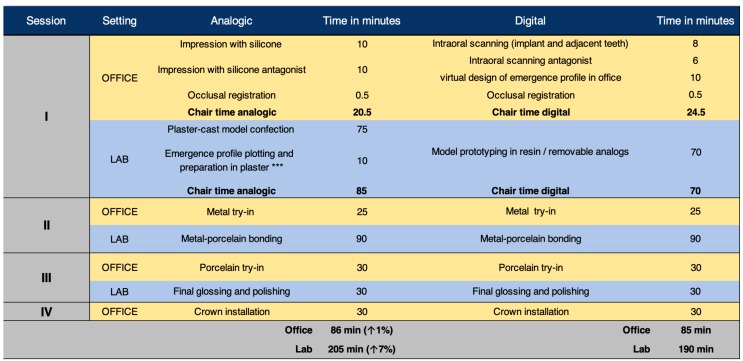
Comparison of chair time consumption between analogical and digital workflows. Abbreviations: ***, if the clinician prefers to make the plotting and preparation of the plaster model in office, the lab should remit the model to the clinic.

From this point, the procedure for preparing and adjusting the BOPT crowns on the resin model (Figure 11) and in the patient (Figure 12 and Figure 13) is similar to that described in the case of the plaster model of the analogical workflow. A scheme is provided depicting time consumption at both office and lab by either analogical and digital workflows; chair time increased around 8% on average with the analogical workflow (Figure 14).

## 5. Discussion

At present, not all the digital procedures have been fully optimized to completely replace the traditional analogical methods. Nevertheless, it is only a matter of time until dental digitalization technology totally replaces the analogical clinical methods [14,15].

The correct development of the digital method in preparing emergence profiles as contemplated in the present study is necessary and inevitable, and its advantages and inconveniences must be compared versus the traditional analogical method.

Analogical preparation of the plaster model through manual reduction and modeling can be done in the laboratory and also in the clinic by dentists who wish to have closer control of this crucial phase of the prosthesis, obtaining the cast and using the rotary/manual instruments available in the dental clinic. Nevertheless, most dentists will find it more convenient to use the digital computer design option prior to obtaining the resin prototype. In this regard, the digital method affords the dentist greater control of these important initial parameters in preparing the BOPT prosthesis in a more simple and effective manner.

The resin prototype has analogs that can be extracted, thereby facilitating the preparation of the prosthesis and the checking of fit or undesired contact zones of the crown upon the working model. However, it must be remembered that in some cases, the fact that these analogs are removable may result in undesired imprecisions compared with the traditional plaster models. Thus, the procedure, the printer quality, the software, and the analogs used in the prototype are crucial factors that must be taken into account [16].

It is also important to remember that the three-dimensional effects when designing the crown contours on the digital model on the computer screen are not as real as when the physical plaster model is used, even to the trained eye [17]. As a result, the emergence profiles initially printed with the prototype model will often require additional minor manual modifications. These can easily be made with rotary instruments by both the dentist and the prosthetic technician in a more “clean and convenient” manner than on the plaster model.

Lastly, it must be remembered that the main inconveniences of the digital method for the dentist very probably are the important efforts needed in terms of economic investment and training associated with the introduction of this technology in the dental clinic. This is particularly so considering that at the present time at least, the increase in quality of the final outcome is not always proportional to the efforts made [18,19,20]. Nevertheless, the “digital approach” has come to stay and we cannot afford delays in adopting this new workflow because things will become increasingly complex for those who “arrive late.” Classical and digital workflows succeeded in giving precise and complete anatomical information of implant position, including the soft tissue contour, and minimal bone loss and aesthetic success could be achieved using both approaches [21]. Moreover, the technology will continue to evolve and will become optimized in the next few years, overcoming the inconveniences which we may still encounter at the present time. 

The advantages of digital flow invite not only dentists but also the work team (e.g., dental hygienist, dental technician, clinic assistants) to engage more active in treatment development through enhancement of communication in the organization and with patients, enriching the knowledge gap on the team. Noteworthy to mention, in both approaches, the final restoration was installed in four visits but an increased chair time consumption is observed with the analogical approach. The average chair time increase was of 8% disaggregated in 1% and 7% for in-office and lab procedures, respectively. Thus, the digital workflow confers slightly better time-effectiveness lower treatment cost. 

The BOPT approach enhances both the supracrestal tissue attachment [8] and peri-implant mucosal sealing through collagen fibers that remain stable and thick over time, with greater efficacy in protecting the supporting bone tissues [5,9,10]. Adequate peri-implant soft tissue environment protects against soft tissue recession and works as a mechanical barrier against food impaction, an inevitable and common problem among patients with an implant-supported prosthesis [22]. Food impaction is a common risk factor for the initiation of peri-implant inflammation and failure of the osseointegrated implants in the long term [23] and distinct to food lodgment that is the mere lodgment of food particles and debris around the peri-implant tissues that can be removed by natural self-cleansing mechanisms [24]. In this sense, long term data of the present approach are warranted in future studies.

## 6. Conclusions

The traditional analogical workflow for the preparation of BOPT prostheses has been extensively developed and affords satisfactory and predictable outcomes. However, the digital approach is undergoing rapid development and optimization, and constitutes the most realistic option, with possibilities for improvement in the immediate future through better communication and data transfer between the dental clinic and the laboratory.
